# Antinociceptive Effect of Aqueous Extract of *Origanum vulgare* L. in Male Rats: Possible Involvement of the GABAergic System

**Published:** 2013

**Authors:** Mohammad Reza Afarineshe Khaki, Yasamin Pahlavan, Gholamreza Sepehri, Vahid Sheibani, Bahare Pahlavan

**Affiliations:** a *School of Medicine, Shahid Beheshti University of Medical Sciences, Tehran, Iran.*; b *Kerman Neuroscience Research Center, Kerman University of Medical Sciences, Kerman, Iran.*; c *Ardabil University of Medical Sciences, Ardabil, Iran. *

**Keywords:** *Origanum vulgare*, Antinociceptive activities, GABAA, GABAB, Muscimol, Bicuculline

## Abstract

The objective of the present investigation was to assess the possible involvement of GABAergic mechanism in analgesic effect of aqueous extract of Origanum Vulgare (ORG) in a rat model of acute pain test. Sixty-three anaesthetized male Wistar rats (200-250 g) were cannulated into the left ventricle. Five to seven days after the recovery from surgery, ORG extract was intraventricularly injected at dose of 3 μg/rat i.c.v. Then, baclofen (10 mg/Kg, IP), CGP_35348_ (100 nmol/Kg, i.c.v), muscimol (1 mg/Kg IP) and bicuculline (5 mg/Kg IP) were separately injected 20 min before the injection of ORG. The experimental groups were compared with intact (control) group (n = 7). The response latency of rats to thermal stimulation was recorded using Tail-Flick test. Injection of ORG extract resulted in a significant and dose-dependent increase in the response latency. There was also a significant increase in the response latency after co-administration of ORG extract with baclofen when compared with control group. However, following co-administration of ORG extract/bicuculline, a significant decrease in the response latency was observed compared to control group. In conclusion, the results of the present study suggest that aqueous extract of *Origanum vulgare L. ssp. viridis *possesses antinociceptive activity in a dose-dependent manner and ORG-induced antinociception might be mediated, at least in part, by both GABA receptors.

## Introduction

Natural products are believed to be an important source of new chemical substances with potential therapeutic application. Several plant species were traditionally used as analgesics (1-3). In general, the determination of herbal plant effects in treatment of disease and pain relief is one of the important strategies in medicine. There are several reports indicating the analgesic effects of medicinal plants (4-7). The present study was designed to evaluate the analgesic effect of *Origanum vulgare L. *in a rat model of acute pain. 

Labiates family of plants are generally known for their multiple pharmacological effects such as analgesic and anti-inflammatory activities (6, 8, 9). *Origanum vulgare *is part of the Labiatae family (10) and belongs to the *Origanum *genus which is native to warm temperate environments from Eurasia to the Mediterranean region (10, 11). ORG is used in traditional medicine as diuretic, stimulant, antimicrobial, anti-inflammatory, antioxidant and anticancer (12). Many of these activities have been attributed to compounds including, carvacrol, thymol, rosmarinic acid, borneol, organol (A and B), ursolic acid, monoterpene hydrocarbons (limonene, terpinene, ocimene, caryophyllene, *β*-bisabolene and p-cymene) and monoterpene alcohols (linalool, 4-terpineol) (13-21).

To the best of our knowledge, analgesic effect of *Origanum vulgare *has not been fully investigated. This work provided evidence of involvement of GABAergic system in ORG-induced analgesic effects in tail flick test.

GABA is the fast-acting inhibitory neurotransmitter in the mammalian brain. It affects different subtypes including GABA_A_ and GABA_B_ receptors (22). It has been shown that the alteration in GABA-mediated inhibition involves in pathological pain (23). This occurs endogenously in animal models of inflammatory and neuropathic pain. In one hypothetical mechanism, the neural transmembrane chloride gradient is distributed after less inhibitory GABAergic input in response to nerve damage (24, 25). Inversely, increasing GABA_A_ receptor-mediated inhibitory alleviates the pain (26). Activation of GABA_A_ receptors can induce strong sedation and other various side effects (27). More of drugs that have natural and herbal origins may not demonstrate these side effects.

## Experimental

*Animals and drugs*

Sixty-three male Wistar rats (200-250 g) were obtained from neurosciences research center of Kerman University of medical sciences. They were housed in light controlled room (12:12 h) and at constant temperature (23 + 3°C) conditions. Animals were fed with standard laboratory diet (Lipton Feed) and water. All of the procedures were in accordance with guidelines for caring and using of laboratory animals in Neuroscience Research Center of Kerman University of Medical Sciences and the Neuroscience Ethic Committee (EC/KNRC/89-5A).

The rats were anesthetized with an intraperitoneal injection of ketamine (80 mg/Kg) / xylazine (10 mg/Kg) mixture and the cannula was stereotaxically inserted into the left brain ventricle according to atlas of Paxinos and Watson (Coordinates from the bregma: AP = - 0.9 mm, DV = - 3.5 mm , ML = - 1.2 mm) (28). The animals were allowed to recover from surgery for 5-7 days prior to behavioral test. Four groups of rats (n = 7) received different doses of extract (1, 3, 6 μg/rat). Same volume of saline (5 μL/rat), as a vehicle, was also intracerebroventricularly injected in saline group. There was also one intact or control group. Thirty min after the injection, the response latency to thermal stimulation was measured 30, 45, 60, 75, 90 and 120 min after drug administration using Tail-Flick test. So, in the primary examination, 3 μg/rat of ORG extract was chosen as an effective dose for the subsequent experiments.

In order to determine the mechanism (s) of the ORG-induced antinociception, rats were divided into 5 groups (n = 7):

a) Saline group (saline 0.5 mL IP/saline 5 μL, i.c.v*.)*

b) Baclofen as a GABA (B) receptor agonist (10 mg/Kg, IP) (29) + ORG group

c) CGP _35348_ as a GABA (B) receptor antagonist (100 nmol/Kg, i.c.v*.*) (30) + ORG group

d) Muscimol as a GABA (A) receptor agonist (1 mg/Kg, IP) (31) + ORG group

e) Bicuculline as a GABA (A) receptor antagonist (5 mg/Kg, IP) (31) + ORG group

The aqueous extract of ORG (3 μg/rat, i.c.v*.*) was administered 20 min after the drug or vehicle injections. The response latency to stimulation of the rat tails was measured 30, 45, 60, 75, 90 and 120 min after the treatment by Tail-Flick test (32). At the end of the experiment, the animals were deeply anesthetized and decapitated. The brain was taken and cannulated accuracy was confirmed.

*Tail flick*

Tail-flick test was used in the present work as described by D’Amour and Smith (33). Briefly, the tail of animal was placed on a level surface, a radiant heat was applied to the tail and the response latency of the rat was recorded to remove its tail from the heat. Here, the maximum time of heat exposure (“cut-off” time) to avoid tissue damage was 10 sec (32). 

*Statistical Analysis*

SPSS software was used for data analysis. Repeated Measurement ANOVA test was used to analysis the data following post test of Bonferroni; p < 0.05 was considered as significant. Group data were shown as mean ± SEM.

## Results and Discussion

The result of this study showed that there was no significant difference in the response latency between control and saline groups [(saline 0.5 mL IP/saline 5 μL, i.c.v*.*) and (saline 5 μL/rat, i.c.v*.*)], so the result of test groups are compared with the control.

**Figure 1:**
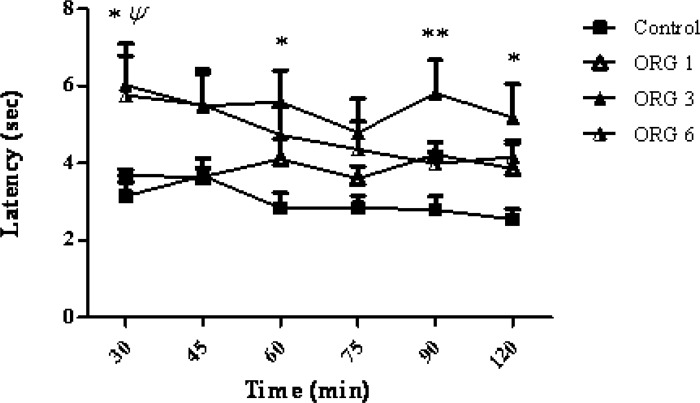
The effect of intracerebroventricular injection of origanum extract (1, 3 and 6 μg/rat) and intact group (control) on tail flick latency of rats. Data represent mean ± SEM of 7 male rats. P-values < 0.05 were considered statistically significant. * = p > 0.05 and ** = p > 0.01, ORG (3 μg/rat) vs control group. ^ψ^* = *p > 0.05, ORG (6 μg/rat) Vs control group.

Intracerebroventricular injection of the ORG extract administered dose-dependently (1, 3 and 6 μg/rat), increased the latency to withdrawal, in the tail-flick test) p < 0.001). A significant difference was observed at a dose of 3 μg/rat of ORG when compared to control group and ORG group (F_3,144_ = 17.17; p < 0.0001). As the Figure 1 shows, the maximum response latency was observed at 30, 60, 90 and 120 min after the ORG injection (p > 0.05, p > 0.05, p > 0.01, p > 0.05, respectively). There was no significant correlation between the response latency and the time after the treatment (F_15,144_ = 0.45, p > 0.0001).

The results of the present study indicate that the aqueous extract of *Origanum vulgare *have markedly central antinociceptive activities in a dose-dependent manner. These data are consistent with the several reports indicating that intraperitoneal injection of aqueous extract of Origanum vulgare can produce a significant analgesic in rats by the formalin test (34). Antinociceptive effect of other species of oregano family such as Origanum onites has also been shown using tail flick test (21). It is reported that the analgesic effect of ORG can be attributed to the existence of the compound named carvacrol. Oregano family of plants is rich in carvacrol (a Terpene Phenyl isomer of Thymol) (21). Carvacrol has an inhibitory effect on prostaglandins. Inhibition of prostaglandins causes antinociception (15). Recently, it has been shown that carvacrol can decrease hypernociception and inflammatory response (14). Oregano is also an important source of rosmarinic acid. Although, some researcher showed that rosmarinic acid could not affect nociceptive response (35), but some researcher showed that this antioxidant compound can produce an analgesic effect in the brain (9).

**Figure 2:**
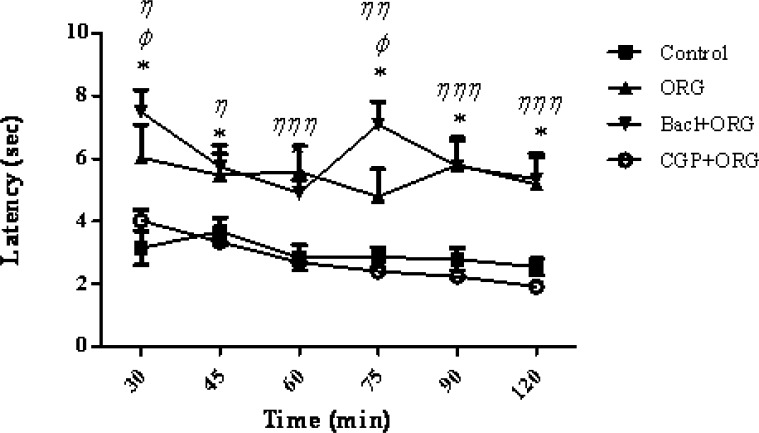
Mean tail flick latencies following the injection of drug or saline in control group, ORG group (ORG 3μg/rat, *i.c.v.*), Baclofen/ORG group and CGP_35348_/ORG group. Data represent mean ± SEM. P-values < 0.05 was considered statistically significant (n = 7). * = p > 0.001, Baclofen/ORG group vs control group. ^Φ ^= p > 0.05, Baclofen/ORG group Vs ORG group. ^η^ = p > 0.05, ^η η^ = p > 0.01 and ^η η η^ = p > 0.001, CGP_35348_/ORG group Vs ORG group.

According to the present results, intraperitoneal injection of baclofen, the GABA B receptor agonist, significantly increased the analgesic effect of intracerebroventricular administration of the ORG extract. There was a significant increase in the response latency or pain threshold after the co-administration of baclofen plus ORG extract as compared to the control group at the times latencies of 30, 45, 60, 75, 90 and 120 min (F_4,180_ = 109.14; Pp 0 .0001) (Figure 1). A significant correlation has been showed between the response latency and the time after treatment (F_20, 180_ = 2.92, p > 0.0001). As it can be seen in Figure 2, CGP _35348_ as a GABA (B) receptor antagonist blocked the analgesia effect of ORG extract. An additional analysis of the results showed that the response latency in (CGP_35348_ / ORG) group was significantly decreased compared with ORG group at 30-120 min after the intervention (p < 0.05). In addition, comparison between baclofen + ORG group and ORG group showed a significant increase in the response latency at 30 and 75 min (p < 0.05) (Figure 2). It has been shown that GABA B-receptors are responsible for the antinociceptive and analgesic effect of baclofen (36). So, the synergist or additive effect of ORG extract and baclofen might be suggested.

**Figure 3:**
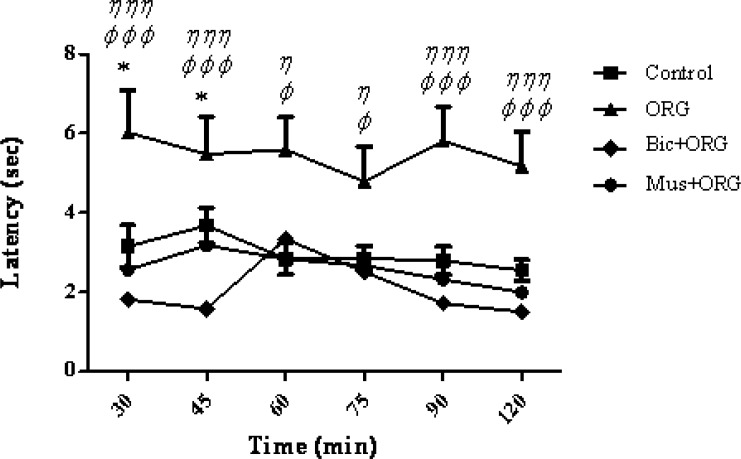
Mean tail flick latencies following the injection of drug or saline in control group, ORG group (ORG 3 μg/rat, i.c.v*.*), Bicuculline/ORG group and Muscimol/ORG group. Data represent mean ± SEM. P-values < 0.05 were considered statistically significant (n = 7). * = p > 0.05, Bicuculline/ORG group Vs control group. ^φ^ = p > 0.05, ^φ φ φ^ = p > 0.001, Bicuculline/ORG group Vs ORG group. η = p > 0.05 and η η η = p > 0.001, Muscimol/ORG group Vs ORG group.

Furthermore, present findings showed a significant decrease in the response latency or pain threshold following the co-administration of bicuculine+ORG compared to control group (at 30 and 45 min. p > 0.05 and p < 0.001, respectively). The combined injection of ORG extract plus muscimol did not show any significant difference when compared to the control group (Figure 3).

The co-administration of muscimol, as a GABA (A) agonist receptor, and ORG extract did not increase the analgesic effect, but bicuculline , as a GABA A receptor antagonist, inhibited the analgesic effect. One possible reason may be the fact that ORG extract and muscimol may induce a competitive antagonist effect on GABA (A) receptors. Therefore, co-administration of muscimol and ORG extract did not produce further effect. An additional analysis of the results showed that the response latency of muscimol/ORG group and bicuculline group showed a significant decrease compared to ORG group at 30-120 min after the intervention (p < 0.05) (Figure 3).

The variable amounts of oxygenated compounds like borneol exist in the leave of origanum (37, 38). Recent studies demonstrated that borneol is a bicycled monoterpene that have analgesic effects and increases the GABA A receptors activity (13). Considering the results of this study, it can be concluded that the analgesic effects of aqueous extract of ORG could be due to the existence of borneol material in aqueous extracts of ORG that induces the stimulation of GABA A receptors.

GABA A receptors are directly connected to the chloride channels and the activity of these receptors directly via opening of chloride channels increase the chloride conductance (39). Bicuculline can antagonize the activity of GABA A receptors (31).

It has also been indicated that the methanolic extract of ORG contains oxygenated compounds such as ursolic acid (40). In-vitro and in-vivo studies have shown that ursolic acid has an anti-inflammatory effect (41, 42).

Finally, the leave of the aqueous extract of origanum vulgare has an antioxidant effect (43) comparable to that of ascorbic acid (11). Rosmarinic acid, carvarol and thymol as the major compounds of ORG extract have effective antioxidant action (11, 18, 44). Other researchers reported that origanol A and origanol B have the antioxidant effects in ORG aqueous extract comparable to those of rosmarinic acid (16, 40). This study demonstrated that the analgesic effect of ORG, at least in part, may be attributed to the effect of ORG extract on GABA receptors (44).

## Conclusion

Findings of the present study showed that the aqueous extract of *Origanum Vulgare L. ssp. *viridis possesses antinociceptive activities in the Tail-Flick test in a dose-dependent manner. In addition, it was indicated that the ORG-induced antinociception may be mediated, at least in part, by GABA receptors activation.
